# Absaugung bei intubierten und tracheotomierten Patient*innen: Eine narrative Übersichtsarbeit

**DOI:** 10.1007/s00101-024-01400-w

**Published:** 2024-04-16

**Authors:** Lars Krüger, Thomas Mannebach, Franziska Wefer, Sarah Lohmeier, Vanessa Stork, Evelin Gosmann, Arnold Kaltwasser

**Affiliations:** 1 Arbeitskreis Evidence-based Nursing (AK EBN), Herz- und Diabeteszentrum NRW, Universitätsklinikum der Ruhr-Universität Bochum, Bad Oeynhausen, Deutschland; 2 Stabsstelle Projekt- und Wissensmanagement/Pflegeentwicklung Intensivpflege, Pflegedirektion, Herz- und Diabeteszentrum NRW, Universitätsklinikum der Ruhr-Universität Bochum, Georgstraße 11, 32345 Bad Oeynhausen, Deutschland; 3 Stabsstelle Pflegeentwicklung, Pflegedirektion, Herz- und Diabeteszentrum NRW, Universitätsklinikum der Ruhr-Universität Bochum, Bad Oeynhausen, Deutschland; 4 Institut für Pflegewissenschaft, Medizinische Fakultät und Universitätsklinik Köln, Universität zu Köln, Köln, Deutschland; 5 Akademie der Kreiskliniken Reutlingen GmbH, Reutlingen, Deutschland

**Keywords:** Atemwegsmanagement, Endotracheale Absaugung, Evidenzbasierte Praxis, Patientensicherheit, Personalschutz, Airway management, Endotracheal suctioning, Evidence-based practice, Patient safety, Staff protection

## Abstract

**Hintergrund:**

Endotracheales Absaugen bei intubierten oder tracheotomierten kritisch kranken Patient*innen fällt in den täglichen Aufgabenbereich verschiedener Berufsgruppen in der Intensiv- und Notfallmedizin.

Eine deutschsprachige Zusammenfassung aktueller Evidenz fehlt bisher.

**Ziel der Arbeit:**

Ziel ist es, eine narrative Übersichtsarbeit über die aktuelle Evidenz zum endotrachealen Absaugen von intubierten oder tracheotomierten Patient*innen im klinischen Setting zu erstellen.

**Material und Methoden:**

Es erfolgten eine Literaturrecherche in den Datenbanken CINAHL, Cochrane Library, LIVIVO, Medline via PubMed, eine Handsuche und ergänzend die Anwendung des Schneeballprinzips durch hochschulisch qualifizierte Pflegende. Eingeschlossen wurden nach erfolgreicher kritischer Beurteilung alle englisch- und deutschsprachigen Publikationen, welche die endotracheale Absaugung im Kontext der Versorgung im Krankenhaus thematisieren.

**Ergebnisse:**

Insgesamt konnten 23 Volltexte eingeschlossen werden, von denen nach der Entwicklung von 6 Oberthemen zur endotrachealen Absaugung 19 in die Berichterstattung aufgenommen wurden.

Im Ergebnis stellte sich u. a. heraus, dass das routinemäßige tiefe Absaugen einmal pro Schicht kontraindiziert ist und der Katheter maximal 0,5–1 cm über das distale Tubus- oder Trachealkanülenende vorgeschoben werden soll. Geschlossene Absaugkatheter bieten, bei allerdings heterogener Studienlage, insbesondere für den Personalschutz Vorteile. Fortbildungen des Personals sind obligat.

**Diskussion:**

Zur endotrachealen Absaugung ließen sich wenige aussagekräftige Studien finden. Mit der vorhandenen Evidenz können jedoch erste Schlussfolgerungen getroffen werden, welche in z. B. internen Standard Operating Procedures berücksichtigt werden sollten. Weitere Forschung ist nötig.

**Zusatzmaterial online:**

Die Online-Version dieses Beitrags (10.1007/s00101-024-01400-w) (siehe QR-Code) enthält die PRISMA 2020 Checkliste und die zugrunde liegende Recherchestrategie.

## Abstract (en)

**Background:**

Endotracheal suctioning in intubated or tracheotomized critically ill patients is a daily task of various professional groups in intensive and emergency medicine; however, a German language summary of current evidence is lacking.

**Objective:**

The aim is to develop a narrative overview of current evidence on endotracheal suctioning of intubated or tracheotomized patients in the clinical setting.

**Material and methods:**

A literature search was conducted in the databases Cinahl, Cochrane Library, Livivo, and Medline via PubMed by nurses with an academic degree. In addition, a hand search and applying the snowball principle were performed. Following a successful critical appraisal, all English and German language publications addressing endotracheal suctioning in the context of hospital care were included.

**Results:**

A total of 23 full texts were included. After developing 6 main topics on endotracheal suction 19 articles were considered in the reporting. The results showed, among others, that routine deep suctioning once per shift is contraindicated and that the catheter should be advanced no more than 0.5–1 cm beyond the distal end of the tube or tracheal cannula. Closed suction catheters offer advantages, especially for staff protection, although studies are heterogeneous. Further training of staff is obligatory.

**Conclusion:**

Few conclusive studies on endotracheal suction could be found; however, with the available evidence initial conclusions can be drawn which should be considered in, for example, internal standard operating procedures. Further research is needed.

## Hinführung zum Thema

Beim endotrachealen Absaugen wird das Sekret aus den Atemwegen mithilfe eines Katheters unter Sog aus der Trachea entfernt. Dies kann entweder über die Nase, einen liegenden nasalen oder oralen Endotrachealtubus (ET), eine Trachealkanüle (TK) oder mittels Bronchoskopie erfolgen. In der klinischen Praxis gibt es unterschiedliche Herangehensweisen beim endotrachealen Absaugvorgang. In dieser Übersichtsarbeit soll die aktuelle Evidenz zum endotrachealen Absaugen über einen ET oder eine TK aufgezeigt werden.

## Hintergrund

Das endotracheale Absaugen von intubierten bzw. tracheotomierten Patient*innen gehört zu den Routinetätigkeiten im Bereich der Intensiv- und Notfallmedizin im Kontext der mukoziliären Clearance. Im multiprofessionellen Team führen neben dem ärztlichen Dienst v. a. auch Pflegende selbstständig Absaugvorgänge durch. Da es sich um einen komplexen invasiven Vorgang mit mannigfaltigen potenziellen Nebenwirkungen und Komplikationen handelt, ist hier die Handlungskompetenz der durchführenden Personen im Sinne der Patient*innensicherheit elementar.

Bei den Patient*innen können Absaugvorgänge vielfältige Symptome auslösen. Dazu gehören beispielsweise Tachykardien, Hypertonien und Änderungen in der Sauerstoffsättigung [2, 13] sowie v. a. auch Ängste und Schmerzen [3, 8]. In den vergangenen Jahren hat die Industrie die zu nutzenden Katheter zur endotrachealen Absaugung weiterentwickelt. Nach ursprünglichen Absaugkathetern mit endständiger Öffnung, welche sich oft an der Trachealschleimhaut festsaugten, folgten Katheter mit Seitenöffnungen, um den Sog bzw. das Vakuum abzuleiten, bis hin zu den 1991 in Deutschland eingeführten geschlossenen Absaugsystemen [16, 25]. Es gibt Hinweise über mögliche Vorteile von geschlossenen Absaugsysteme zum Erhalt der Beatmungsparameter [20] und zum Personalschutz [17].

Für die klinische Praxis fehlt es im deutschsprachigen Raum an einer Übersicht zur aktuellen Evidenz beim endotrachealen Absaugen. Kaltwasser und Dubb [15] entwickelten einen evidenzbasierten Handlungsalgorithmus zur endotrachealen Absaugung, welcher v. a. auf den Empfehlungen der American Association for Respiratory Care (AARC) von 2010 beruht. Eine Überarbeitung dieser Empfehlungen wurde 2022 publiziert [6], die z. T. neue Erkenntnisse auflistet. Überdies gab es entsprechend weitere Forschungsarbeiten.

Ziel dieser Arbeit ist es, eine Übersicht über die aktuelle Evidenz zum endotrachealen Absaugen von Patient*innen mithilfe des ET oder der TK im klinischen Setting zu erstellen.

## Methode

Die Übersichtsarbeit orientiert sich an den Empfehlungen der „Preferred Reporting Items for Systematic Reviews and Meta-Analyses (PRISMA) reporting guideline“ [23] (Zusatzmaterial online: die zugrunde liegende PRISMA_2020_Checklist).

Es erfolgte eine Literaturrecherche durch hochschulisch qualifizierte Pflegende mit mindestens einem Jahr Berufserfahrung auf der Intensivstation im Rahmen ihrer Tätigkeit innerhalb des Arbeitskreises Evidence-based Nursing (AK EBN) an einem Universitätsklinikum. In einem gemeinsamen Abstimmungsprozess wurden dazu zunächst die Suchbegriffe für die Datenbankrecherchen festgelegt und mit den Booleschen Operatoren AND/OR verknüpft. Eine Verknüpfung mit NOT fand nicht statt. Die Literaturrecherche wurde am 13.09.2021 in den Datenbanken CINAHL, Cochrane Library, LIVIVO und Medline via PubMed durchgeführt. Je nach Datenbank variierte die Suchstrategie, welche im Zusatzmaterial online: ESM 2 abgebildet wird. Eine Aktualisierung der Literaturrecherche fand am 09.05.2023 statt (Zusatzmaterial online: die Recherchestrategie).

Ergänzend wurde weitere Literatur nach dem Schneeballprinzip anhand der Literaturverzeichnisse recherchiert. Darüber hinaus erfolgte eine Handsuche in einer medizinischen Bibliothek und einer Weiterbildungsstätte.

Eingeschlossen wurden alle englisch- und deutschsprachigen Publikationen, die im Kontext der Versorgung im Krankenhaus stehen und die endotracheale Absaugung thematisieren. Nichtrelevante Publikationen wurden zunächst durch Titel- und Abstractscreening ausgeschlossen. Anschließend erfolgte eine kritische Bewertung der Volltexte mit den Beurteilungsbögen von Behrens und Langer [5]. Final eingeschlossen wurden Publikationen mit einer Benotung ≤ 3. Die Volltexte wurden anschließend analysiert und systematisch in einer strukturierten Tabelle (Autor, Jahr, Studiendesign, Setting/Population, Intervention, Outcome-Parameter, Ergebnisse und Qualität der Studie) zusammengefasst. Der gesamte Prozess fand unter fortlaufender Beratung durch eine hochschulisch qualifizierte Pflegefachperson mit ausgewiesener Expertise für das Thema endotracheale Absaugung statt.

## Ergebnisse

**Figure 1:**
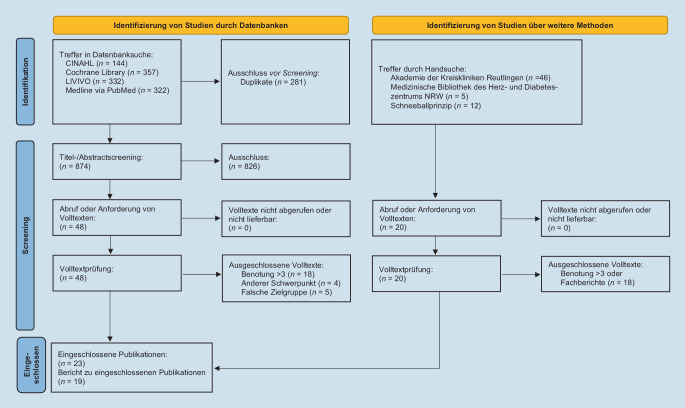

Insgesamt konnten 23 Volltexte eingeschlossen werden (Abb. 1).

Aus den eingeschlossenen Volltexten wurden aufgrund der Limitation in den Referenzen 19 in diese Arbeit aufgenommen. Dazu zählen 4 systematische Reviews [11, 12, 26, 28], 3 randomisierte kontrollierte Studien (RCT) [4, 8, 19], eine Pilot-RCT [29], eine Cross-over-Studie [27], eine kontrollierte Studie [2], 2 Beobachtungsstudien [9, 13], eine Querschnittstudie [31], eine Evaluationsstudie [22], eine retrospektive Datenanalyse [14] sowie 4 Praxisleitfäden und Empfehlungen [6, 10, 15, 17]. Sechs Publikationen beziehen sich primär auf pädiatrische Patient*innen [9, 11, 19, 27–29]. Für die Berichterstattung erfolgte die Auflistung von 6 praxisrelevanten Oberthemen, zu denen die Inhalte narrativ zusammengefasst wurden.

### Indikation zur endotrachealen Absaugung

Das Vorhandensein eines ET oder einer TK in Verbindung mit maschineller Beatmung verhindert die physiologische Atemwegs-Clearance und macht das Absaugmanöver notwendig.

Die endotracheale Absaugung sollte grundsätzlich nur bei erkennbar vorhandenem Sekret erfolgen, mit den Zielen, angesammeltes Lungensekret zu entfernen, die Atemwege offen zu halten und somit die Oxygenierung sicherzustellen [6, 10]. Allgemein ist ein protokollgestütztes Vorgehen sinnvoll [4, 8]. Anzeichen für Sekretverlegungen sind [6, 15, 19]:

- Patient*in meldet Bedarf an,
- akute Atemnot,
- Atemgeräusche oder auskultatorische Rasselgeräusche,
- Verringerung des Atemzugvolumens bei druckkontrollierter Beatmung,
- Anstieg des inspiratorischen Spitzendruckes bei volumenkontrollierter Beatmung,
- sichtbares Sekret im künstlichen Atemweg,
- Abfall des S_p_O_2_ und/oder Verschlechterung der Blutgaswerte,
- zusätzliche radiologische Befunde,
- Verdacht auf Aspiration von Sekreten aus dem Magen oder den oberen Atemwegen,
- Entnahme von Bronchialsekret zur mikrobiologischen/pathologischen Untersuchung,
- Sägezahnmuster auf der Flusskurve des Beatmungsgerätes,
- akuter Anstieg des Atemwegswiderstandes.

Da losgelöst von der Sekretentfernung ET und TK verlegt werden können, sollte alle 8 h eine Durchgängigkeitsprüfung in Form eines Absaugvorgangs mit Vorschieben des Absaugkatheters bis zum distalen Ende des ET bzw. der TK durchgeführt werden [6].

### Kontraindikation zur endotrachealen Absaugung

Der Absaugvorgang sollte nur bei Bedarf durchgeführt werden [6, 19]. Ist das Absaugen indiziert, gibt es keine absoluten Kontraindikationen. Die meisten relativen Kontraindikationen beziehen sich auf das Risiko des Erleidens von Komplikationen, welche beim Prozess entstehen und Auswirkungen auf den klinischen Zustand der Patient*innen haben können [6]. Komplikationen im Kontext der endotrachealen Absaugung sind u. a. Hypoxie, Arrhythmien, kardiovaskuläre Instabilität, Atelektasenbildung, mikrobakterielle Kolonisation der unteren Atemwege, Verletzungen von Gewebsstrukturen mit ggf. Blutungen, Schmerzen und Erhöhung des intrakraniellen Druckes [6, 22].

### Hygienemaßnahmen beim Absaugvorgang

Die hygienisch korrekte Durchführung der endotrachealen Absaugung über einen ET oder eine TK ist ein wichtiger Aspekt in der Prävention von nosokomialen Pneumonien. Diese werden nach einer Beatmungsdauer von mindestens 48 h als beatmungsassoziiert („ventilator-associated pneumonia“, VAP) bezeichnet [17].

**Figure 2:**
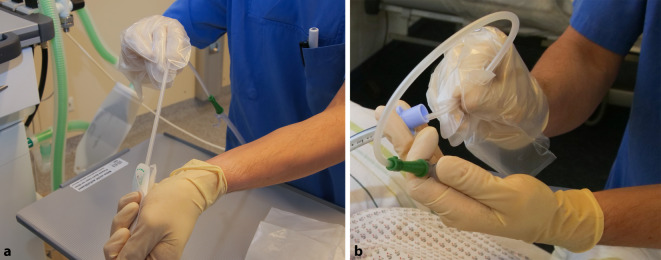

Zur Vorbereitung einer grundsätzlich aseptisch durchzuführenden endotrachealen Absaugung [6] gehören neben der hygienischen Händedesinfektion eine wischdesinfizierte Ablagefläche, das Anlegen einer persönlichen Schutzausrüstung (PSA) in Form eines Mund-Nasen-Schutzes, unsteriler und steriler Einweghandschuhe sowie ggf. einer Schutzbrille [10, 15]. Überdies ist es wichtig, dass die Patient*innen, sofern möglich, ihre Augenlider schließen oder diese entsprechend abgedeckt werden, um aerosolbedingte Infektionen am Auge zu vermeiden [1]. Die „Peel-off“-Technik zur Öffnung der sterilen Verpackung findet im Zuge einer offenen Absaugung Anwendung, um die Kontamination des Katheters zu vermeiden. Dieser kann dann nach Überstreifen eines sterilen Einweghandschuhs in den ET oder die TK eingeführt werden. Die nichtkatheterführende Hand entfernt dabei die Schutzhülle und übernimmt die unsterilen Tätigkeiten (Abb. 2a, b).

Dieser Vorgang kann auch mit der Unterstützung/Assistenz einer zweiten Fachperson stattfinden. Moderne Respiratoren verfügen über entsprechende Präoxygenierungs- bzw. Absaugunterstützungsprogramme, welche bei Diskonnexion des Beatmungssystem zusätzlich den Atemgasfluss unterbrechen und dabei einen Kontaminationsschutz des Personals und der Umgebung unterstützen. Bei einem hygienisch kritisch zu sehenden offenen Absaugvorgang über einen Konnektor am ET oder an der TK darf die maschinelle Beatmung nicht unterbrochen werden.

Das geschlossene Absaugsystem zeichnet sich durch einen in das Beatmungssystem integrierten, steril verpackten, wiederverwendbaren Absaugkatheter aus, welcher eine möglichst keimarme Durchführung ermöglicht [6, 17]. Bezüglich der positiven Effekte auf eine nachweisbare nosokomiale Pneumonie unter maschineller Beatmung bildet sich eine heterogene Studienlage ab [4, 6, 12–14, 17, 26]. Grebe und Hering [12] konnten beispielsweise in einem systematischen Review einen leicht positiven, jedoch statistisch nicht signifikanten Effekt (Relatives Risiko [RR] 0,81, 95 %-Konfidenzintervall [95 %-KI] 0,63; 1,03) zugunsten des geschlossenen Absaugsystems, bezogen auf die Entstehung einer VAP, identifizieren. Blakeman et al. [6] fassten hierzu zusammen, dass die meisten Studien keine statistisch signifikanten Unterschiede ausmachen konnten.

Die Nutzung eines geschlossenen Absaugsystems ist v. a. dann indiziert, wenn Patient*innen mit multiresistenten Erregern (MRE) in den Atemwegen kolonisiert sind. Eine Exposition des Personals oder eine potenzielle Kontamination durch unbeabsichtigten Kontakt mit der Patient*innenumgebung ist in diesem Fall nahezu ausgeschlossen [6, 10, 15, 17]. Die Kommission für Krankenhaushygiene und Infektionsprävention beim Robert Koch-Institut (RKI; KRINKO) [17] verweist hierzu u. a. auf eine ältere Untersuchung von Cobley et al. [7] mit kleiner Stichprobe (*n* = 7), in der die Kolonisation der Umgebungsluft während des Absaugvorgangs (offen vs. geschlossen) gemessen wurde. Im Ergebnis konnte in der durchschnittlichen Mittelwertdifferenz (MD) von 18,6 (offen: 25,3 vs. geschlossen: 6,7) gezählten Kolonien ein statistisch signifikanter Unterschied (*p* = < 0,001) zugunsten des geschlossenen Absaugsystems festgestellt werden.

Geschlossene Absaugsysteme werden gemäß den Herstellendenangaben gewechselt. Allgemein sollte dies nach 48 h [17] oder durch spezifische Regelungen in einer einrichtungsinternen Standard Operating Procedure (SOP) erfolgen.

### Auswahl von Absaugkathetern und Absaugsystemen

Bei der Auswahl des Absaugkatheters müssen neben den hygienischen Aspekten weitere Punkte beachtet werden [6]. Zum einen ist es wichtig, dass der Außendurchmesser des Katheters dem Innendurchmesser (ID) des ET angepasst wird. Ein zu großer Katheter lässt sich aufgrund der Reibung im ET oder in der TK schlecht vorschieben. Er könnte außerdem Verletzungen oder Schäden an den Schleimhäuten verursachen, wenn es zu einem weiten Vorschieben über das distale Ende des ET oder der TK kommt. Überdies könnte es bei Okklusion in einer Bronchiole oder falscher Beatmung zu einem Unterdruck in den Alveolen kommen. In jedem Fall muss durch das verbleibende „Restlumen“ eine Beatmung oder Spontanatmung weiterhin möglich bleiben. Hierzu wird empfohlen, dass der Außendurchmesser des Katheters bei pädiatrischen und erwachsenen Patient*innen < 50 % des ID beträgt [6, 8]. Als Berechnungsgrundlage kann hierzu eine Eins vom ID des ET oder der TK subtrahiert und das Ergebnis verdoppelt werden [24]. Als Gesamtergebnis wird dann der passende Absaugkatheter in Charrière (Charr) angezeigt; eine Einheit, welche im Englischen synonym auch als French (F) bezeichnet wird.

Beispiel zur Errechnung des passenden Absaugkatheters für einen ET mit ID von 8 mm:

$$(8-1)\times 2=14\,\text{Charr}$$

Das Ergebnis zeigt, dass bei einem ET oder einer TK mit einem ID von 8 mm ein Absaugkatheter mit 14 Charr passend ist.

Der Einsatz von geschlossenen Absaugkathetern wird losgelöst von den hygienischen Gesichtspunkten seit Jahren diskutiert und kann bei erwachsenen und pädiatrischen Patient*innen Komplikationen verhindern. Dazu gehören die Vermeidung von Hypoxien sowie die Erhaltung des positiv-endexspiratorischen Drucks („positive endexpiratory pressure“, PEEP) und der Kreislaufstabilität [2, 8, 12, 18, 21, 27, 28]. In einer kontrollierten Studie von Afshari et al. [2] wurden beispielsweise u. a. kardiorespiratorische Parameter (mittlerer arterieller Blutdruck [MAD], Herzfrequenz [HF] und arterielle Sauerstoffsättigung) als Outcome beim offenen im Vergleich zum geschlossenen Absaugen vor und zu verschiedenen Zeitpunkten nach dem Absaugvorgang fokussiert. Im Ergebnis veränderte sich vor und 1 Min nach dem Absaugvorgang im Mittelwert der MAD (von 82,6 auf 85,9 vs. 81,1 auf 82,2), die HF (von 84,8 auf 95,3 vs. 84,0 auf 87,5) und die arterielle Sauerstoffsättigung (von 97,7 auf 93,2 vs. 98,0 auf 97,3). Über alle ermittelten Werte hinweg konnten bei der HF (*p* = 0,025) und arteriellen Sauerstoffsättigung (*p* = 0,001) im Gegensatz zum MAD (*p* = 0,287) eine statistisch signifikante Abweichung zugunsten des geschlossenen Absaugsystems festgestellt werden.

Bezogen auf HF, Blutdruck und Sauerstoffsättigung wurden ebenso bei pädiatrischen Patient*innen positive und z. T. statistisch signifikante Effekte [9, 27, 28], jedoch innerhalb einer Pilotstudie auch keine klinisch relevanten Unterschiede beider Methoden [29] festgestellt. Evans et al. [9] heben bei der offenen im Vergleich zur geschlossenen Absaugung zusätzlich eine statistisch signifikant verringerte Vorbereitungs- bzw. Absaugzeit (38 vs. 23 Min/Tag; *p* = < 0,001) hervor. Insgesamt hält die Autorenschaft der AARC-Leitlinie fest, dass sowohl offene als auch geschlossene Absaugsysteme Trachealsekret effizient entfernen [6]. Yilmaz und Ozden [30] verglichen in einer randomisierten kontrollierten Cross-over-Studie das offene mit dem geschlossenen Absaugen. Die Autorenschaft fand u. a. heraus, dass es bezüglich der Entfernung der Sekretmenge im Mittelwert (5,438 g/Tag vs. 5,197 g/Tag) keinen statistisch signifikanten Unterschied (*p* = 0,675) zwischen beiden Systemen gab. Die Studienlage zu entstehenden Kosten für beide Absaugsysteme ist heterogen und von verschiedenen Faktoren wie z. B. Nutzungsdauer und Personalschutz abhängig [8, 17, 26].

### Präoxygenierung, Absaugtiefe, Sogstärke und Lavage

Eine generelle Präoxygenierung vor dem Absaugvorgang wird empfohlen und kann eine absaugbedingte Hypoxämie verhindern [6]. Zeitlich werden dafür 30–60 s [15] bis hin zu 180 s [10] angegeben. Präoxygenierungsprogramme von modernen Respiratoren unterstützen diesen Prozess und lassen teilweise eine Auswahl der Sauerstoffbeimischung zu.

**Figure 3:**
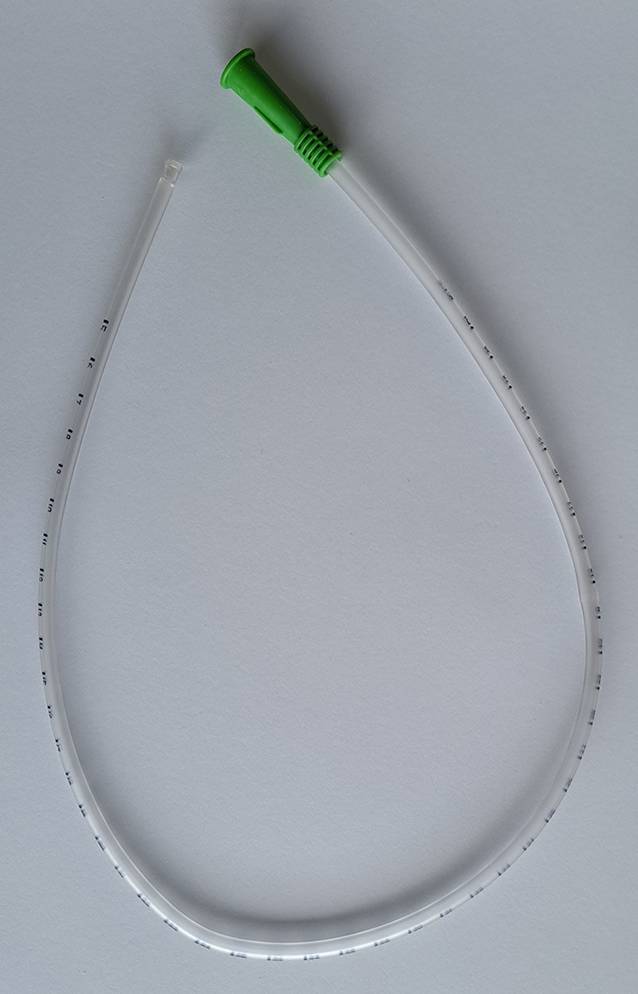

Eine Absaugtiefe von max. 0,5–1 cm über das distale Ende des ET/der TK [6, 13] sollte bei Routineabsaugmanövern eingehalten werden. Bei Neugeborenen und Kleinkindern konnten Gillies und Spence [11] in einem Cochrane-Review jedoch keine statistisch signifikanten Unterschiede zwischen einem tiefen und flachen Absaugen feststellen (HF 141,2 vs. 137,5; MD 3,68; 95 %-KI −8,34; 15,70). Dennoch sollte ein tiefes Absaugmanöver nur stattfinden, wenn das flache Absaugmanöver ineffizient und das Potenzial für negative Auswirkungen, wie z. B. Traumata, gering ist [6]. Eine Graduierung am Absaugkatheter ermöglicht in der Praxis ein zielgenaues Platzieren desselben. Bei geschlossenen Systemen ist dies regelhaft vorhanden; offene Absaugkatheter können ebenfalls mit Graduierung erworben werden (Abb. 3).

Als Absaugdauer sind 10 bis max. 15 s einzuhalten [6, 13, 19]. Die Sogeinstellung soll so niedrig wie möglich und < 0,25 bar bei erwachsenen sowie < 0,15 bar bei pädiatrischen Patient*Innen sein [6, 13, 15, 32]. Der Einsatz einer Lavage mit Kochsalzlösung während des Absaugmanövers ist als obsolet zu betrachten und bleibt der Bronchoskopie vorbehalten [6, 15].

### Fortbildung und Schulungen

Yilmaz et al. [31] untersuchten die Kenntnisse und Erfahrungen von türkischem Intensivpflegepersonal mit dem geschlossenen Absaugsystem. Im Ergebnis berichteten 71,8 % der Befragten keine Herausforderungen beim Absaugen mit einem geschlossenen System, 85,6 % gaben hierzu Zeitersparnis an. Rund die Hälfte (50,8 %) der Teilnehmenden berichteten jedoch, dass die Entfernung von zähflüssigem Sekret für sie problematisch sei. Die Autorenschaft schlussfolgerte, dass kontinuierliche Fort- und Weiterbildung zum Thema Absaugen für Pflegende obligat ist, um Handlungskompetenzen aufzubauen [31].

Negro et al. [22] befragten Pflegende auf 16 Intensivstationen in italienischen Krankenhäusern zu den Inhalten der AARC-Leitlinie von 2010. Im Ergebnis konnten hier 2,5 % der Pflegenden 9 von insgesamt 10 Fragen korrekt beantworten. Die Autorenschaft kam auch hier zu dem Schluss, dass eine gute Ausbildung und entsprechende (Fort‑)Bildungsprogramme zum endotrachealen Absaugen benötigt werden [22].

Für Deutschland verweist die KRINKO dazu auf § 23 Abs. 8 Nr. 5 IfSG sowie Hygieneverordnungen der Bundesländer, nach denen u. a. Fortbildungen zur Infektionsprävention für das betreffende Personal obligat sind [17]. An dieser Stelle ist für Pflegende auch auf das Pflegeberufegesetz (PflBG) zu verweisen. § 5 Abs. 1, S. 2 listet hier das lebenslange Lernen, welches als elementar angesehen wird, auf.

## Diskussion

Diese Übersichtsarbeit hatte zum Ziel, die aktuelle Evidenz zur endotrachealen Absaugung bei Patient*Innen mit einem ET oder einer TK im klinischen Setting abzubilden. Aus insgesamt 19 identifizierten Volltexten erfolgte eine narrative Zusammenfassung zu 6 Oberthemen.

Die vor vielen Jahren oft praktizierte tägliche routinemäßige tiefe Absaugung von Patient*innen mithilfe eines ET oder einer TK ist kontraindiziert und sollte in der klinischen Praxis nicht durchgeführt werden [6]. Lediglich eine Kontrolle der Durchgängigkeit des Device im Zeitfenster von je 8 h ist wichtig, um Komplikationen zu vermeiden [6]. Hierzu soll der Absaugkatheter jedoch, anders als beim endotrachealen Absaugvorgang zur Sekretentfernung, max. bis zum distalen Ende des ET bzw. der TK vorgeschoben werden.

Es sollten Absaugkatheter verschiedener Größen, die dem aktuellen industriellen Standard nach dem Deutschen Institut für Normung (DIN-Norm) entsprechen, vorgehalten werden, um sie je nach ID des vorhandenen Zugangs auswählen zu können. Ein zu großer Katheter scheint subjektiv zunächst effektiver abzusaugen, kann jedoch gefährliche Komplikationen verursachen [6, 24]. Geschlossene Absaugkatheter sind dem offenen Absaugen nicht per se vorzuziehen, scheinen aber Vorteile in Bezug auf Kontaminationsschutz [17], den reduzierten Zeitaufwand [9] sowie die Vermeidung von potenziellen Komplikationen zu bieten [12, 27]. Generell ist die Studienlage hierzu aber noch heterogen. Durch den verbesserten Personalschutz beim geschlossenen Absaugsystem sollte grundsätzlich diskutiert werden, ob mögliche Mehrkosten das geeignete Argument für ein offenes Absaugverfahren sind.

Eine Prä- und Postoxygenierung sowie das Einhalten einer Maximaldauer sind beim endotrachealen Absaugen empfehlenswert [15], eine Lavage bleibt jedoch der Bronchoskopie vorbehalten [6]. Hierzu muss kritisch erwähnt werden, dass in manchen Studien eine Lavage mit Kochsalzlösung zum Einsatz kam [9, 27], die jedoch nicht empfohlen wird [6]. Inwiefern dies einen Einfluss auf die jeweiligen Ergebnisse hatte bleibt offen. Generell soll ein „flaches“ gegenüber dem „tiefen“ Absaugen mit eher niedrigen Sogstärken bevorzugt werden [6].

Der endotracheale Absaugvorgang ist unabhängig von der Art des Zugangs oder der verwendeten Katheter ein komplexer und potenziell komplikationsbehafteter Prozess, der einer umfassenden und fortlaufenden Schulung und Expertise des durchführenden Personals bedarf [22]. Vor allem in Bezug auf erworbenes bzw. vorhandenes Wissen von Pflegenden zum endotrachealen Absaugen zeigen sowohl Yilmaz et al. [31] als auch Negro et al. [22] Lücken auf, die es in der Praxis zu schließen gilt. Auch wenn die Ergebnisse nicht 1:1 auf die Situation in Deutschland übertragbar sind, bedarf es intensiv geschulter Multiplikator*Innen und einer Aufnahme des Themas in Einarbeitungsprozesse aller Berufsgruppen, die eine endotracheale Absaugung durchführen.

Für ein derart wichtiges und komplexes Thema ließen sich überraschenderweise nur vergleichsweise wenige wirklich aussagekräftige Studien finden. Grundsätzlich können mit der bisher vorhandenen Evidenz zwar mit einiger Zuversicht Schlussfolgerungen getroffen werden, anhand derer z. B. interne SOP angepasst und aktualisiert werden sollten. Insbesondere in Bezug auf offenes vs. geschlossenes Absaugen im Kontext der VAP ist aber noch weitere Forschung nötig. Da bisher nur international eine englischsprachige Leitlinie identifiziert werden konnte [6], wäre eine solche auch für den deutschsprachigen Raum wünschenswert.

## Limitationen und Stärken

In dieser Übersichtsarbeit wurden ausschließlich Publikationen berücksichtigt, die bei der kritischen Beurteilung nach Behrens und Langer [5] mit einer Benotung von ≤ 3 bewertet wurden. Weitere Volltexte hätten ggf. zusätzliche Evidenz einbringen können, wurden jedoch als nicht ausreichend glaubwürdig eingeschätzt. Eine Recherche in weiteren Datenbanken wie z. B. CareLit, die primär Fachartikel ohne ein Peer-Review-Verfahren auflisten, fand nicht statt. Hier hätten ebenso weitere Erkenntnisse generiert werden können. Der gesamte Prozess von der Entwicklung der Suchstrings über die Sichtung und Bewertung bis hin zur Auswertung erfolgte durch hochschulisch qualifizierte Pflegende. Die Generierung der Oberthemen fand praxisnah statt. Inhaltlich konnten jedoch für die Fachpraxis nicht alle relevanten Punkte umfassend abgebildet werden.

## Schlussfolgerung

Das evidenzbasierte Handeln wird u. a. von Pflegenden und dem ärztlichen Dienst seit jeher gefordert. Die Ergebnisse dieser Übersichtsarbeit können zu einer evidenzbasierten Praxis beitragen und in einrichtungsinterne SOP, Einarbeitungsprozesse oder die Überarbeitung von Handlungsalgorithmen aufgenommen werden. Pflegende mit einem Hochschulabschluss können (z. B. innerhalb eines AK EBN) zur Entwicklung einer evidenzbasierten Praxis beitragen. Die Kombination aus grundlegenden Kenntnissen und Fähigkeiten im wissenschaftlichen Arbeiten sowie der intensivpflegerischen Fachpraxis und Einbindung einer Pflegefachperson mit ausgewiesener Expertise im Bereich der endotrachealen Absaugung erwies sich hierzu als sehr hilfreich.

## Fazit für die Praxis

- Pflegende, der ärztliche Dienst und weitere Berufsgruppen, die Absaugvorgänge durchführen, sollten sich zum endotrachealen Absaugen kontinuierlich fort- und weiterbilden.
- Für die endotracheale Absaugung sind Absaugkatheter zu verwenden, deren Außendurchmesser < 50 % des ID des ET bzw. der TK entspricht.
- Die endotracheale Absaugung sollte, losgelöst von der Durchgängigkeitsprüfung, nur bei erkennbar vorhandenem Sekret erfolgen.
- Beim Absaugvorgang sollte protokollgestützt vorgegangen werden.
- Bei Absaugvorgängen sollte der Absaugkatheter nicht mehr als 0,5–1 cm über das distale Ende des ET bzw. der TK vorgeschoben werden.
- Die Auswirkungen einer geschlossenen Absaugung auf die Entwicklung einer VAP sind bisher nicht mit einer aussagekräftigen Evidenz hinterlegt.
- Geschlossene Absaugsysteme haben Vorteile im Bereich des Personalschutzes und sollen bei MRE-Kolonisation eingesetzt werden.
- Eine generelle Präoxygenierung sollte vor jedem Absaugvorgang stattfinden.
- Als Absaugdauer sind 10 bis max. 15 s einzuhalten.

### Supplementary Information






## References

[1] Aci (2021). Agency for Clinical Innovation (ACI). Agency for Clinical Innovation (ACI).

[2] Afshari A, Safari M, Oshvandi K (2014). The effect of the open and closed system suctions on cardiopulmonary parameters: time and costs in patients under mechanical ventilation. Nurs Midwifery Stud.

[3] Anbeh T (2002). Psychologische Aspekte einer Intensivstation: : Studie über beatmete Langzeitpatienten.

[4] Ardehali SH, Fatemi A, Rezaei SF (2020). The Effects of Open and Closed Suction Methods on Occurrence of Ventilator Associated Pneumonia; a Comparative Study. Arch Acad Emerg Med.

[5] Behrens J, Langer G (2010). Evidence-based Nursing and Caring. Methoden und Ethik der Pflegepraxis und Versorgungsforschung.

[6] Blakeman TC, Scott JB, Yoder MA (2022). AARC Clinical Practice Guidelines: Artificial Airway Suctioning. Respir Care.

[7] Cobley M, Atkins M, Jones PL (1991). Environmental contamination during tracheal suction. A comparison of disposable conventional catheters with a multiple-use closed system device. Anaesthesia.

[8] Dastdadeh R, Ebadi A, Vahedian-Azimi A (2016). Comparison of the Effect of Open and Closed Endotracheal Suctioning Methods on Pain and Agitation in Medical ICU Patients: A Clinical Trial. Anesth Pain Med.

[9] Evans J, Syddall S, Butt W (2014). Comparison of open and closed suction on safety, efficacy and nursing time in a paediatric intensive care unit. Aust Crit Care.

[10] Fiebig T (2017). Endotracheales Absaugen – Schritt für Schritt. Krankenhhyg up2date.

[11] Gillies D, Spence K (2011). Deep versus shallow suction of endotracheal tubes in ventilated neonates and young infants. Cochrane Database Syst Rev.

[12] Grebe M, Hering T (2018). Closed versus open endotracheal suctioning and the risk of Ventilator-associated Pneumonia. Systematic Review and Metaanalysis. PflWiss.

[13] Jongerden IP, Kesecioglu J, Speelberg B (2012). Changes in heart rate, mean arterial pressure, and oxygen saturation after open and closed endotracheal suctioning: a prospective observational study. J Crit Care.

[14] Juneja D, Javeri Y, Singh O (2011). Comparing influence of intermittent subglottic secretions drainage with/without closed suction systems on the incidence of ventilator associated pneumonia. Indian J Crit Care Med.

[15] Kaltwasser A, Dubb R (2021). Endotracheal suctioning. Med Klin Intensivmed Notfmed.

[16] Kaltwasser A, Dubb R, Hekler M (2001). Endotracheales Absaugen. Handbuch der Intensivpflege Ecomed.

[17] Krinko (2013). Prävention der nosokomialen beatmungsassoziierten Pneumonie. Empfehlung der Kommission für Krankenhaushygiene und Infektionsprävention (KRINKO) beim Robert Koch-Institut. Bundesgesundheitsblatt Gesundheitsforschung Gesundheitsschutz.

[18] Kuriyama A, Umakoshi N, Fujinaga J (2015). Impact of closed versus open tracheal suctioning systems for mechanically ventilated adults: a systematic review and meta-analysis. Intensive Care Med.

[19] Lema-Zuluaga GL, Fernandez-Laverde M, Correa-Varela AM (2018). As-needed endotracheal suctioning protocol vs a routine endotracheal suctioning in Pediatric Intensive Care Unit: A randomized controlled trial. Colomb Med.

[20] Maggiore SM, Lellouche F, Pigeot J (2003). Prevention of endotracheal suctioning-induced alveolar derecruitment in acute lung injury. Am J Respir Crit Care Med.

[21] Maggiore SM, Lellouche F, Pignataro C (2013). Decreasing the adverse effects of endotracheal suctioning during mechanical ventilation by changing practice. Respir Care.

[22] Negro A, Ranzani R, Villa M (2014). Survey of Italian intensive care unit nurses’ knowledge about endotracheal suctioning guidelines. Intensive Crit Care Nurs.

[23] Page MJ, Moher D, Bossuyt PM (2021). PRISMA 2020 explanation and elaboration: updated guidance and exemplars for reporting systematic reviews. BMJ.

[24] Pedersen CM, Rosendahl-Nielsen M, Hjermind J (2009). Endotracheal suctioning of the adult intubated patient—what is the evidence?. Intensive Crit Care Nurs.

[25] Sackner MA, Landa JF, Greeneltch N (1973). Pathogenesis and prevention of tracheobronchial damage with suction procedures. Chest.

[26] Solà I, Benito S (2007). Closed tracheal suction systems versus open tracheal suction systems for mechanically ventilated adult patients. Cochrane Database Syst Rev.

[27] Taheri P, Asgari N, Mohammadizadeh M (2012). The effect of open and closed endotracheal tube suctioning system on respiratory parameters of infants undergoing mechanical ventilation. Iran J Nurs Midwifery Res.

[28] Taylor JE, Hawley G, Flenady V (2011). Tracheal suctioning without disconnection in intubated ventilated neonates. Cochrane Database Syst Rev.

[29] Tume LN, Baines PB, Guerrero R (2017). Pilot Study Comparing Closed Versus Open Tracheal Suctioning in Postoperative Neonates and Infants With Complex Congenital Heart Disease. Pediatr Crit Care Med.

[30] Yilmaz I, Ozden D (2024). The effects of open and closed system endotracheal suctioning methods on suctioning frequency, amount of secretion, and haemodynamics: A single-blind, randomised, 2 x 2 crossover trial. Aust Crit Care.

[31] Yilmaz I, Ozden D, Arslan GG (2021). Intensive care nurses’ evidence-based knowledge and experiences regarding closed suctioning system. Niger J Clin Pract.

[32] Yousefi H, Vahdatnejad J, Yazdannik AR (2014). Comparison of the effects of two levels of negative pressure in open endotracheal tube suction on the physiological indices among patients in intensive care units. Iran J Nurs Midwifery Res.

