# Supplementary material for: Suctioning in intubated and tracheotomized patients: A narrative review
Source: Anaesthesiologie. 2024 Apr 16;73(5):340–7. [Article in German] doi: 10.1007/s00101-024-01400-w (PMC11076389; doi:10.1007/s00101-024-01400-w)
Supplement: Supplementary file 2 [file 101_2024_1400_MOESM2_ESM.pdf]

Zusatzmaterial zum Beitrag **„Absaugung bei intubierten und tracheotomierten Patient*innen“** von Lars Krüger, Thomas Mannebach, Franziska Wefer et al. (2024) in *Die Anaesthesiologie*
Beitrag und Zusatzmaterial stehen Ihnen auf www.springermedizin.de zur Verfügung. Bitte geben Sie dort den Beitragstitel in die Suche ein.

Recherchestrategie

CINAHL	TX suction* AND TX ((close* OR open)) AND TX ((hospital or acute setting or inpatient or ward)) AND TX ((breath* OR ventilation OR "mechanical ventilation"))
Cochrane Library	#1 suction<br>#2 close* OR open<br>#3 hospital OR clinic<br>#4 adult* OR child<br>#5 breath* OR ventilation OR "mechanical ventilation"<br>#6 #1 AND #2 AND #3 AND #4<br>[Search manager]
Livivo	((suction* AND (close OR open)) AND (hospital OR clinic)) AND (breath* OR ventilation OR "mechanical ventilation") [Open search]
Medline via PubMed	(suction*) AND (close* OR open) AND (hospital OR clinic) AND (breath* OR ventilation OR "mechanical ventilation") [All Fields]

Aktualisierung am 09.05.2023
